# An automated approach to identify scientific publications reporting pharmacokinetic parameters

**DOI:** 10.12688/wellcomeopenres.16718.1

**Published:** 2021-04-21

**Authors:** Ferran Gonzalez Hernandez, Simon J Carter, Juha Iso-Sipilä, Paul Goldsmith, Ahmed A. Almousa, Silke Gastine, Watjana Lilaonitkul, Frank Kloprogge, Joseph F Standing

**Affiliations:** 1 CoMPLEX, University College London, London, UK; 2 The Alan Turing Institute, London, UK; 3 Institute of Pharmacy, Uppsala University, Uppsala, Sweden; 4 Institute for Global Health, University College London, London, UK; 5 BenevolentAI, London, UK; 6 Eli Lilly and Company, London, UK; 7 London Health Sciences Centre, Ontario, Ontario, Canada; 8 Great Ormond Street Institute of Child Health, University College London, London, UK; 9 Institute of Health Informatics, University College London, London, UK; 10 Health Data Research, London, UK

**Keywords:** Information extraction, Pharmacokinetics, Natural Language Processing, Machine Learning, Bioinformatics, Text mining, Pharmacometrics

## Abstract

Pharmacokinetic (PK) predictions of new chemical entities are aided by prior knowledge from other compounds. The development of robust algorithms that improve preclinical and clinical phases of drug development remains constrained by the need to search, curate and standardise PK information across the constantly-growing scientific literature. The lack of centralised, up-to-date and comprehensive repositories of PK data represents a significant limitation in the drug development pipeline.In this work, we propose a machine learning approach to automatically identify and characterise scientific publications reporting PK parameters from in vivo data, providing a centralised repository of PK literature. A dataset of 4,792 PubMed publications was labelled by field experts depending on whether in vivo PK parameters were estimated in the study. Different classification pipelines were compared using a bootstrap approach and the best-performing architecture was used to develop a comprehensive and automatically-updated repository of PK publications. The best-performing architecture encoded documents using unigram features and mean pooling of BioBERT embeddings obtaining an F1 score of 83.8% on the test set. The pipeline retrieved over 121K PubMed publications in which in vivo PK parameters were estimated and it was scheduled to perform weekly updates on newly published articles. All the relevant documents were released through a publicly available web interface (https://app.pkpdai.com) and characterised by the drugs, species and conditions mentioned in the abstract, to facilitate the subsequent search of relevant PK data. This automated, open-access repository can be used to accelerate the search and comparison of PK results, curate ADME datasets, and facilitate subsequent text mining tasks in the PK domain.

## Introduction

Recent studies estimate the average cost for a drug to reach marketing approval at $1.3 billion. Meanwhile, success rates after Phase I clinical trials lie below 15% ^1^. Early and precise prediction of a drug candidate’s properties represents a crucial aspect for improving the efficiency of drug development. Over the last decade, mathematical modelling and bioinformatics have emerged as fundamental tools for model-informed decision-making in drug discovery and development ^2^. Despite the recent advances in computational methods, the scarcity of standard protocols and centralised repositories still hinder drug discovery and development from exploiting the full potential of quantitative approaches.

One area of particular relevance is in the pharmacokinetic (PK) profiling of new drug candidates. Although mechanistic models (e.g. physiologically-based PK) have been extensively used to predict *in vivo* PK properties (i.e. Absorption, Distribution, Metabolism and Excretion (ADME)) of new chemical entities, a large proportion of drug candidates are still discarded due to PK complications detected at the clinical phases ^3– 5^. Therefore, improving PK predictions at an early stage represents a critical task to better evaluate new candidate prospects and optimise the drug development pipeline.

One of the main barriers to improve *in vivo* PK predictions of new compounds is the availability of large, diverse and centralised PK data of approved drugs ^6, 7^. Preclinical predictions of PK parameters (primarily: absorption rate, bioavailability, systemic clearance, volume of distribution and elimination half-life) of a newly discovered drug are largely based on prior knowledge from other compounds, which is often obtained from a variety of sources. The most structured and readily available source of PK data is from chemical databases. In the public domain, several databases have been developed and maintained to provide detailed information on a large number of molecules. Some of the main open-access databases that store chemical descriptors and PK data include DrugBank ^8^, PubChem ^9^, ChEMBL ^10^ and ACToR ^11^. Despite the extensive structural and physicochemical information stored in these databases, the amount and detail of *in vivo* PK data is often very sparse ^7^. For instance, PK parameters are usually reported for a specific population (e.g. healthy adults) and a single administration route (e.g. intravenous). Additionally, very limited information about the study design or modelling approach from which PK parameters were estimated is readily available. As a result, before preclinical PK predictions can be made, additional data needs to be collated from unstructured sources.

Most of the datasets used for preclinical PK predictions (so-called ADME datasets) aggregate and combine data from manually curated in-house studies, publicly available databases and information extracted from the scientific literature ^12, 13^. For instance, the PK/DB database ^4^ collected information from public databases and the scientific literature, providing fine-grained PK information that can be used for preclinical PK predictions. Specifically, parameter values, demographics, study design and sampling information was stored from over 1203 compounds at PK/DB. Despite the high-quality data curated in PK/DB, this aggregation becomes a time-consuming task since it requires researchers to manually search, curate and standardise PK data from multiple sources before predictive analyses can be performed. Additionally, manual curation limits our ability to cope with the vast and constantly increasing biomedical literature. For instance, PubMed ^14^ comprises over 30 million citations, increasing at a rate of approximately two papers per minute ^15^, which makes the task of identifying relevant studies both complex and time-consuming.

The other area of relevance is population PK model building, which remains laborious, computationally intensive and requires expert input. Recent attempts to automate parts of this process include using genetic algorithms to search for optimal covariates and model configurations ^16, 17^ although, on the flip side, the computational burden increases even further. Automated PK model development could benefit from prior information already out in the literature to achieve improved efficiency. However, despite recent initiatives ^18^, most of the population PK models remain locked in the scientific literature.

Text mining approaches have been applied to deal with the extensive and in-coming scientific literature and address the limitations of manual data curation. In the PK domain, very few studies have applied text mining to process scientific publications. However, a few studies have addressed related challenges for biochemical kinetic systems (e.g. enzyme kinetics), which often report parameters from ordinary differential equations ^19– 21^. In the PK domain, Wang *et al.* ^7^ applied rule-based approaches to extract numerical values referring to systemic and oral clearance after administration of midazolam in healthy human volunteers. The highly specific task addressed by Wang *et al.* ^7^ made dictionary and rule-based approaches particularly suited to filter irrelevant abstracts and maximise the precision of the information extracted. This approach aimed to detect a single PK parameter in a specific context (intravenous/oral midazolam administration to healthy human volunteers). However, the viability and time required to adopt this approach for various PK parameters and contexts (other species, conditions, drugs) remain unclear.

The type of PK information required to construct ADME datasets is likely to depend on each study’s end-goal. To develop comprehensive PK repositories that accelerate ADME dataset curation, it is essential to extract multiple PK parameters (e.g. clearance, bioavailability, half-life, volume of distribution) and their estimated values from multiple drugs. Additionally, for a specific compound, different administration routes (e.g. oral, intravenous), species (e.g. humans, mice, pigs), or specific modelling approaches (e.g. compartmental/non-compartmental) should be considered. This diversity represents a significant limitation for text mining approaches, which exclusively rely on rules and dictionaries that need to be handcrafted for the specific context on a case by case basis. In contrast, if sufficient high-quality training data are available, machine learning (ML) approaches can be particularly suited to account for this diversity and model highly complex rules.

A PubMed search for "pharmacokinetics" (January 2021) returns over half a million entries. Despite the broad coverage of this search, only ...20% (See Table 1) of the resulting articles estimate PK parameters from *in vivo* data. Hence, when constructing ADME datasets, comparing PK results across papers, or searching for prior information for PK modelling, researchers need to efficiently filter a large amount of irrelevant literature. To overcome the limitations of manual curation and rule-based approaches, we developed an ML pipeline to identify scientific publications reporting PK parameters from *in vivo* data. Additionally, we characterised relevant PK studies by the drugs, species and conditions mentioned in the respective abstracts to accelerate the search and comparison of PK results. Finally, this resource was made publicly available through a web interface at https://app.pkpdai.com/.

## Methods

Due to the heterogeneous literature and the need for a high-quality corpus of PK papers, a supervised ML approach was applied to identify scientific publications reporting *in vivo* PK parameters. The specific steps performed involved:

1. **Corpus development:** manual annotation of scientific articles depending on whether they reported *in vivo* PK parameters.
2. **Pipeline development:** comparison of different pipeline architectures to optimise the classification of relevant publications.
3. **Large-scale application:** application of the best-performing pipeline to retrieve and characterise a large collection of PK papers.

All our code and models were released at https://github.com/PKPDAI/PKDocClassifier ^22^.

### Corpus development

***Source.*** Due to the large size and broad coverage of the PubMed search "pharmacokinetics", a protocol was developed to filter for relevant entries within this collection of papers. A corpus of documents was manually labelled to train and evaluate different classification pipelines. The PubMed search "pharmacokinetics" was performed without further filtering criteria, and the resulting list of PubMed identifiers was downloaded (October 2020). All the publications labelled in this study were selected by simple random sampling without replacement using the python library *random* .

***Size and criteria.*** To train, compare and evaluate different classification approaches two collections of documents were developed: the *training* and the *final test* sets. The *training* and *final test* sets consisted of 3992 and 800 randomly-sampled articles, respectively. Each set’s size was determined based on the availability of annotators, ensuring at least two independent labels per document. The articles were labelled according to the following criteria: If a particular publication reported newly estimated PK parameters obtained *in vivo* in either the title, abstract, tables or full-text, it was labelled as *Relevant* ^1^. Publications without PK parameter estimates, reviews or articles mentioning PK parameters from other studies were considered *Not Relevant*. Only the original publications in which PK parameters were estimated together with their contextual information were labelled as *Relevant*.

***Annotation.*** The annotation process was carried out by two clinical pharmacists and two pharmacometricians from the Pharmacometrics Group at University College London and one clinical pharmacist from the London Health Sciences Center, all with extensive training in pharmacometric modelling. Each document in the *training* set was initially labelled by two annotators, and at least three annotators initially labelled the documents from the *final test*. Disagreements were subsequently reviewed with all the annotation team. Exceptions and conflicting opinions on the labelling criteria emerged during labelling, and, after resolution of each case, guidelines were updated accordingly. To evaluate the agreement across annotators, the Cohen Kappa Coefficient ( *K*, Equation 1) was initially calculated on 100 documents sampled from the test set which were labelled by five annotators ^23^. *K* compares the observed agreement between two annotators (p *_o_*) and the agreement expected by chance (p *_e_*) on the true class ( *Relevant* documents):

$$K=\frac{p_{o}-p_{e}}{1-p_{e}}\;(1)$$

During the labelling procedure, guidelines were iteratively updated until a high consistency between annotators was obtained ( *K*>0.9).

### Pipeline development

***Data retrieval.*** Since full-text and tabular information are not always readily available, only the textual information from the title, abstract and other PubMed metadata were used to train the classification pipelines. These fields are publicly available for most papers. The development of a retrieval system without utilising the tables and full-text information makes the pipeline applicable to a broader range of publications. The desired documents were downloaded in XML format from the official PubMed baseline and updatefiles FTP sites and parsed with PubMed Parser ^24^.

### Evaluation

**Figure 1.**
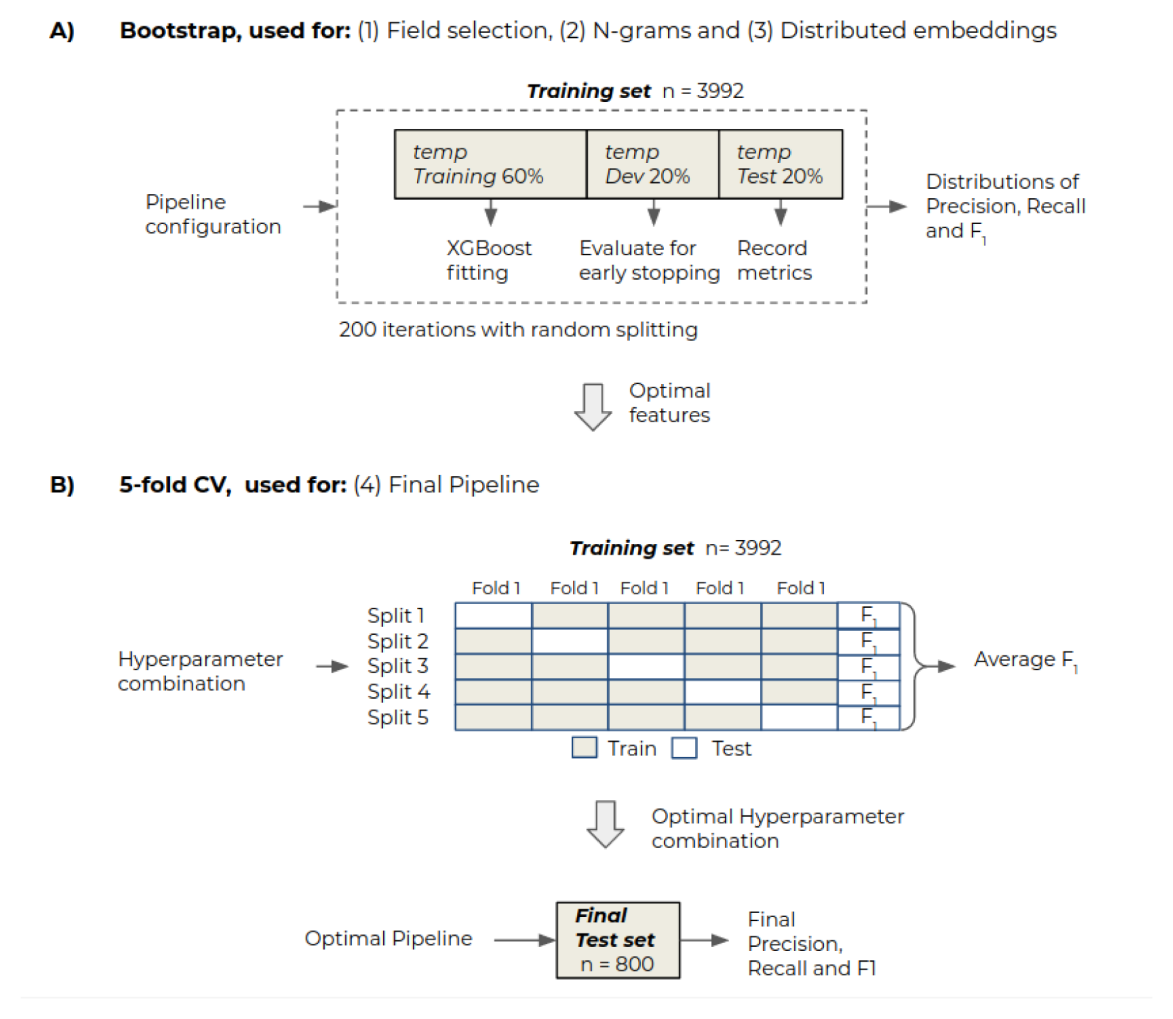
**A**) Bootstrap procedure to compare the effect of different features during field selection, n-grams and distributed representations analyses. **B**) The best-performing features from previous analyses were selected to compare different hyperparameter combinations with 5-fold cross validation. Finally, the best-performing features and hyperparameters were used to apply the pipeline to the final test set.

The goal of the pipeline development stage was to maximise the classification performance of *Relevant* papers by investigating the effects of different pipeline configurations. Analyses were performed to assess the effect of the input textual fields, document representations and the pipeline hyperparameters ^2^. To compare the different architectures, precision (P), recall (R) and *F*1 score (the harmonic mean between precision and recall) were used as evaluation metrics. To select the best-performing architecture, we performed analysis on (1) Field selection, (2) N-grams, (3) Distributed representations and (4) Final pipeline. The objective of analyses 1, 2 and 3 was to detect the best-performing document representations for the classification task. Due to the limited number of training samples and associated variability on *F*_1_, a bootstrapping approach was employed to obtain a distribution of metrics for each pipeline configuration ( Figure 1 A). To avoid bias on the evaluation, at each iteration of bootstrapping, the *training set* was randomly split into *temp training* (60%), *temp dev* (20%) and *temp test* (20%) with stratified sampling using scikit-learn’s python module. The *temp training* set was used to fit the classifier, *temp dev* to evaluate the classifier during training and perform *early stopping* and *temp test* to record the pipeline performance after each bootstrap iteration. This process was repeated 200 times for each pipeline configuration to obtain a distribution of metrics. Once the best-performing document representations were detected, the whole *training set* was used to select the best-performing hyperparameters, and this was finally applied to the *final test set* to obtain the final metrics ( Figure 1B).

### Classifier

Extreme Gradient Boosting (XGBoost) was employed as the classifier in all the analyses. XGBoost is an optimised implementation of Gradient Boosting developed by Chen *et al.*, 2016 ^25^ that exhibited excellent performance in multiple ML applications ^26– 28^ due to improved regularisation techniques that highly reduce overfitting and effectively handle sparse data ^29^.

Boosting is an ensemble method that builds a strong model *H _L_* ( *x* ) as a weighted sum of *L* weak learners *f _l_* ( *x* ):

$$H_{L}(x)=\sum_{l=1}^{L}\eta \cdot w_{l}\cdot f_{l}(x)\;(2)$$

Where *w _l_* is the weight associated with the new weak learner and *η* (learning rate) controls each weak learner’s contribution by a constant value. New weak learners *f _l_* ( *x*) are sequentially added to the ensemble until the maximum number of iterations is reached. In gradient boosting, a new weak learner is fitted to the negative gradient of the loss function in each iteration ^30^. Decision trees were used as weak learners over which the XGBoost was trained to minimise the average cross-entropy loss as the objective function. To address potential prediction biases due to class imbalance, the weights associated with each sample during the training stage were set to be inversely proportional to the class frequency on the corpus. In other words, if only 20% of documents in the training set were labelled as *Relevant*, the loss associated with each *Relevant* sample was scaled by a factor of five. This loss weighting protocol effectively prevented any potential spurious solutions where predictions may be biased towards the most frequent class (e.g. more *Not Relevant* than *Relevant* papers in the training set). Finally, early stopping was employed where the maximum number of iterations was set to 2000 and boosting was stopped earlier if the *F*_1_ performance on the validation ( *temp dev*) set did not improve after 100 iterations. This approach was used to prevent overfitting by limiting the number of boosting iterations through an external evaluation ( *temp dev* set) and, in turn, limiting the complexity of the model.

**1. Field selection**

Fields extracted from the PubMed metadata included: *authors*, *journal*, *publication type*, *keywords*, *MeSH terms* ^3^, *chemicals* ^4^ and *affiliations*. Table 1 summarises the availability of these fields in our corpus. The abstract’s importance was initially assessed by comparing the performance when only using the title against the combination of title + abstract. Subsequently, each field’s relevance in the metadata section was studied by comparing the performance when using each field in combination with the title and abstract. Finally, fields that exhibited an increase in the classification performance were combined.

**Table 1.** Summary statistics reporting the percentage of documents in which a particular field was available, and the proportion of papers labeled as Relevant and Not Relevant. The statistics are reported for both training and final test sets.

Field	Training	Final test
Title	100	100
Abstract	87.17	87.67
Authors	99.44	99.63
Journal	100	100
Publication Type	100	100
Keywords	15.41	16.125
MeSH terms	97.67	98.25
Chemicals	93.86	94.13
Affiliations	80.94	79.125
Label		
Relevant	19.81	20.25
Not Relevant	80.19	79.75

For the field selection analyses, documents were encoded using a Bag-of-Words (BoW) approach, which represented documents as fixed-length vectors based on their term frequencies ^32^. Each unique term in the corpus received an *id* that corresponded to a position in the document vector representation of size “n” (n = vocabulary size). Given an input document, each cell in the document vector was filled with the frequency of that term, and the resulting vector was divided by the total number of terms in that document (L1 vector norm). Before applying the BoW, each document was segmented into semantic units ( *tokens*) ^33^. Meta-data fields from PubMed were treated as single tokens without further pre-processing. For the *title* and *abstract* text, the rule-based tokeniser from scispaCy ^34^ was used, which is specifically designed to tokenise scientific text. The *title* and *abstract* tokens were lower-cased, punctuation signs were removed from inside the tokens, and those tokens entirely composed by digits were replaced with the same token (##). Additionally, stop-words were removed to reduce the vocabulary size. Chemical mentions were detected with scispaCy’s named entity recogniser (NER) ^5^ ^34^ and replaced by the same token to prevent bias towards specific chemical mentions.

Finally, the Porter’s stemmer algorithm ^35^ was applied to each token to standardise related word forms.

**2. N-grams**

Since the BoW model does not consider any order in the sequence of input words, all tokens are treated as independent features. The *n-gram* approach aims to encapsulate some sequential information by generating groups of tokens (of size *n*) that appear sequentially in the text. For this analysis, the *optimal* fields from *field selection* were used, and the effect of adding *bigrams* and *trigrams* from the abstract and title as additional features was studied.

**3. Distributed representations**

Instead of representing documents with sparse vectors, distributed representations refer to dense, fixed-sized vectors of much lower dimensionality than the vocabulary size, which encode semantic and contextual information of a particular word, sentence or document. These representations are often learnt by pre-training deep neural networks on large unlabelled corpora. Bidirectional Encoder Representations from Transformers (BERT) ^36^ has achieved state-of-the-art results on a wide variety of natural language processing (NLP) tasks (e.g. sentence classification, language inference, NER, question answering). In this study, BERT was employed to generate distributed representations of scientific documents. BERT learns powerful word representations by using a bi-directional Transformer ^37^ as an encoder and pretraining the model with “masked language model” and “next sentence prediction” objectives ^36^. In this analysis, two models based on BERT were used as feature extractors: BioBERT and SPECTER.

**Figure 2.**
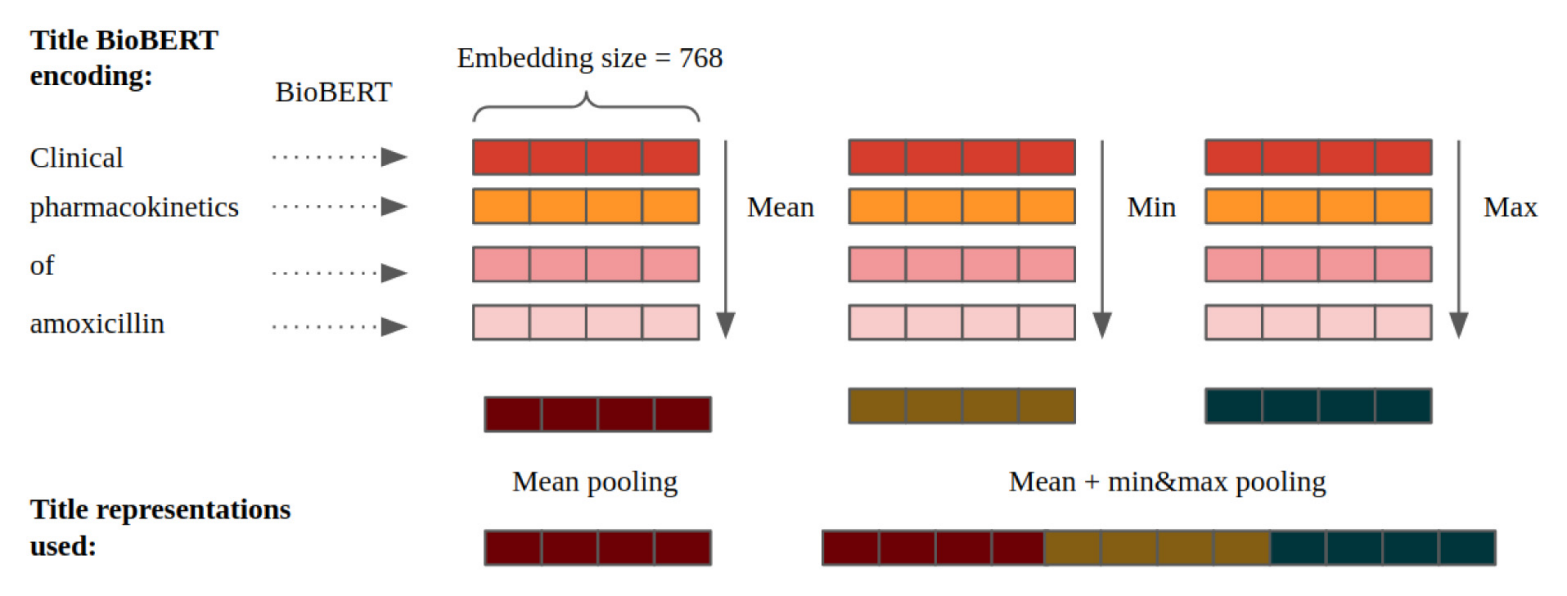
Example of the approaches used to generate distributed representations for an input title after BioBERT encoding. The same procedure was applied for tokens in the abstract.

**BioBERT** Lee *et al.*, 2019 ^38^ developed BioBERT by further training the BERT-Base model on PubMed abstracts and PMC full-text articles to learn word representations in the biomedical domain. In this study, distributed token representations were obtained by adding the last four hidden layers of BioBERT, producing 768-dimensional vectors for each token. For a given document, its representation was obtained by concatenating the BioBERT encodings of the title and the abstract. Since the number of tokens differed across documents, a composition function was applied to combine multiple token vectors into fixed-length document representations. For this, two approaches were compared, illustrated in Figure 2. First, the mean across all token representations was computed ( *mean pooling*), which resulted in 1536-dimensional document representations (768 from the title + 768 from the abstract). Then, the minimum and maximum ( *min&max pooling*) were computed and concatenated with *mean pooling* producing 4608-dimensional document representations.

**SPECTER** Instead of using token-level representations, Cohan *et al.*, 2020 ^39^ proposed a BERT-based approach to generate document-level representations for scientific articles directly. SPECTER pre-trains the Transformer to encode scientific documents using the title and abstract to learn closer representations for related publications. The pre-training objective of SPECTER consists on predicting whether specific articles are cited by a central input document. During inference, SPECTER encodes the abstract and title of each input publication into a single 768-dimensional vector used as a representation for the whole document. The authors reported state-of-the-art performance on seven document-level tasks ^39^. Here, the classification performance was also studied when using document representations from SPECTER as input features.

**4. Final pipeline**

The best-performing features from the previous analyses were combined into a single model to determine whether they provided complementary information. Then, the hyperparameters of the pipeline were adjusted using a five-fold cross-validation (CV) approach with exhaustive grid search on the whole *training set* ( Figure 1B). For the implementation of the grid-search, a number of candidate values were initially specified for each hyperparameter. The hyperparameters tuned included:

1. *min_df* : Minimum number of documents that a specific token should appear to be included in the BoW feature matrix. This hyperparameter has a large impact on the size of the feature vectors generated by BoW.
2. *max_depth*: Maximum depth of each decision tree build in the boosting process.
3. *colsample_bytree*: Proportion of features that each decision tree subsamples at each boosting iteration.
4. *n_estimators*: Number of boosting iterations.

The range of values specified for each hyperparameter is shown in Table 2. During the *field selection*, *n-grams* and *distributed representations* analyses, only *n_estimators* was optimised during training whilst all the other hyperparameters were kept constant at their default values ( Table 2). The XGBoost learning rate was kept constant at 0.1. The rest of XGBoost hyperparameters were kept constant to their default values in the scikit-learn API.

**Table 2.** Hyperparameters tuned during cross-validation and their default values. The range represents the different values tested for each hyperparameter in the grid-search procedure. The step size refers to the increase between the starting and stop values.

Parameter	Range (start, stop, step)	Default value
*min_d f*	(2,512,x2)	20
*max_depth*	(2,64,x2)	4
*colsample_bytree*	(1/3,1,+1/3)	1
*n_estimators*	Early stopping	-

### Large-scale application

Once the final pipeline was trained, it was applied to classify over 550K papers that were obtained from the PubMed search on “pharmacokinetics”. The final pipeline was implemented in Apache Spark and deployed through Azure Databricks ^40^. The retrieved documents were characterised by the chemicals, diseases and species mentioned in the abstract using the BERN algorithm ^41^. BERN uses a fine-tuned version of BioBERT to perform NER and normalisation of drug names, species, diseases, mutations and genes. A detailed description of the finetuning procedure and standardisation in BERN is provided in Donghyeon *et al.*, 2019 ^41^.

## Results and discussion

### Inter-annotator agreement

For the 100 randomly-sampled articles, the initial pairwise *K* was 0.68 *±* 0.073 (mean *±* standard deviation) ^22^. The initial disagreement was mostly observed due to: (1) eventual missing of *Relevant* instances by the annotator, (2) differences in the labelling criteria of edge cases, e.g. pharmacokinetic studies of endogenous substances, physiologically-based PK studies and (3) cases where PK parameters were not reported in the abstract, and the full-text was not accessible. Disagreements of type 1 were easily detected through double annotations and posterior checking. To reduce disagreements due to the labelling criteria (type 2), guidelines were iteratively updated through discussions with the annotation team until the inter-annotator agreement exceeded a pairwise *K* of 0.9. Finally, the most complex cases were those in which the full-text was not available, and the abstract was not clear on whether PK parameters were estimated in the study (type 3). For those cases, exhaustive checks between the whole annotation team were performed, and the final label was assigned based on the most common criteria across annotators.

### Field selection

**Figure 3.**
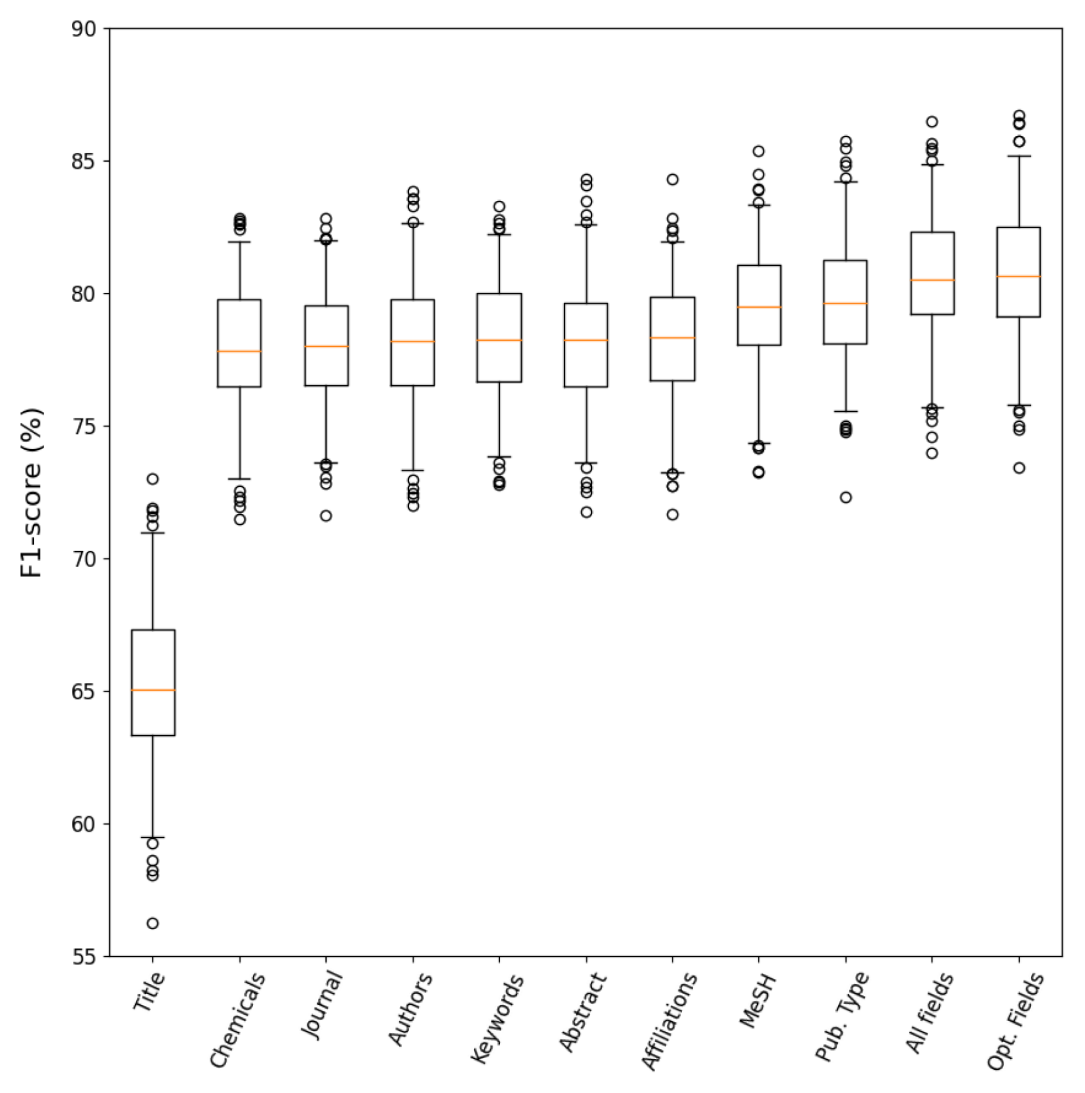
Distribution of *F*_1_ scores for the different features used in the *field selection* analysis after 200 bootstrap iterations. The fields Chemicals, Journal, Authors, Keywords, Affiliations, MeSH terms and Publication Type were encoded together with the title and abstract tokens. The Optimal Fields include the title, abstract, MeSH terms and Publication Type.

Evaluation metrics were computed after 200 bootstrapping iterations on the *training set* to evaluate each PubMed field’s importance for the classification task. The results from this analysis are displayed in Table 3 and Figure 3. It was observed that using information from the abstract in combination with the title (Abstract pipeline, median *F*_1_=78.2%) provided a great advantage compared to only using the title (Title pipeline, median *F*_1_=65%). Additionally, a number of fields did not provide any discriminant information when added to the title and abstract: *chemicals*, *journal*, *authors*, *keywords*. It can be noticed that adding *affiliations* had a very small increase in the median *F*_1_ score ( *∆F*_1_=0.1%) but no clear improvement could be observed from the distribution of *F*_1_ scores ( Figure 3). On the other hand, adding the *MeSH terms* and *Publication Type* as additional fields exhibited a clear improvement in the distribution of *F*_1_ scores with over 1% gain in the median *F*_1_ in comparison to only using the abstract and title. The *Publication Type* field had particular relevance to determine whether PK publications were Reviews containing PK information from other studies, which helped to detect *Not Relevant* publications with similar word frequencies to the *Relevant* documents.

**Table 3.** Summary table with performance metrics reported as median (95% CI) and *F*_1_ interquartile variance (IQV) after 200 bootstrap iterations. The performance metrics are compared across pipelines using different fields from PubMed entries.

Pipeline	Precision (%)	Recall (%)	*F*_1_ (%)	*F*_1_ IQV
Title	65.3 (59.0,72.7)	65.8 (55.0,72.8)	65.0 (59.5,71.0)	11.5
Abstract	77.0 (69.8,82.6)	79.8 (73.4,86.1)	78.2 (73.6,82.6)	9.0
Authors *	76.4 (69.4,82.6)	80.4 (72.8,86.1)	78.2 (73.3,82.6)	9.3
Journal *	76.4 (70.2,82.2)	79.8 (72.8,85.4)	78.0 (73.6,82.0)	8.4
Publication Type *	78.0 (71.6,84.3)	81.6 (74.7,87.4)	79.6 (75.5,84.2)	8.7
Keywords *	76.6 (70.2,83.0)	80.4 (72.8,85.5)	78.2 (73.8,82.2)	8.4
MeSH terms *	79.2 (72.1,85.2)	79.8 (72.8,86.1)	79.5 (74.3,83.3)	9.0
Chemicals *	76.0 (69.5,81.9)	80.4 (73.4,86.1)	77.8 (73.0,82.0)	9.0
Affiliations *	76.6 (69.8,82.1)	80.4 (72.8,86.7)	78.3 (73.2,81.9)	8.7
All fields	**80.1 (73.0,86.1)**	81.6 (74.1,87.4)	80.5 (75.7,84.9)	9.2
Optimal Fields **	**80.1 (73.9,86.0)**	**82.3 (74.1,88.6)**	**80.6 (75.8,85.2)**	9.4

*Tokens from the title and abstract were also included when encoding this field. **The optimal fields were the title, abstract, MeSH terms and publication type.

Given the previous observations, the *Title*, *Abstract*, *MeSH terms* and *Publication Type* were considered to be the *Optimal Fields*. Despite the large number of field combinations that could be explored, only using the optimal fields exhibited a similar (and slightly higher) performance than the pipeline using all the fields ( Table 3). Hence, a significant reduction in the number of features was obtained without loss in the classification performance by only using the *Optimal Fields*. In subsequent analyses, only the *Optimal Fields* were considered for BoW encoding.

### N-grams

**Figure 4.**
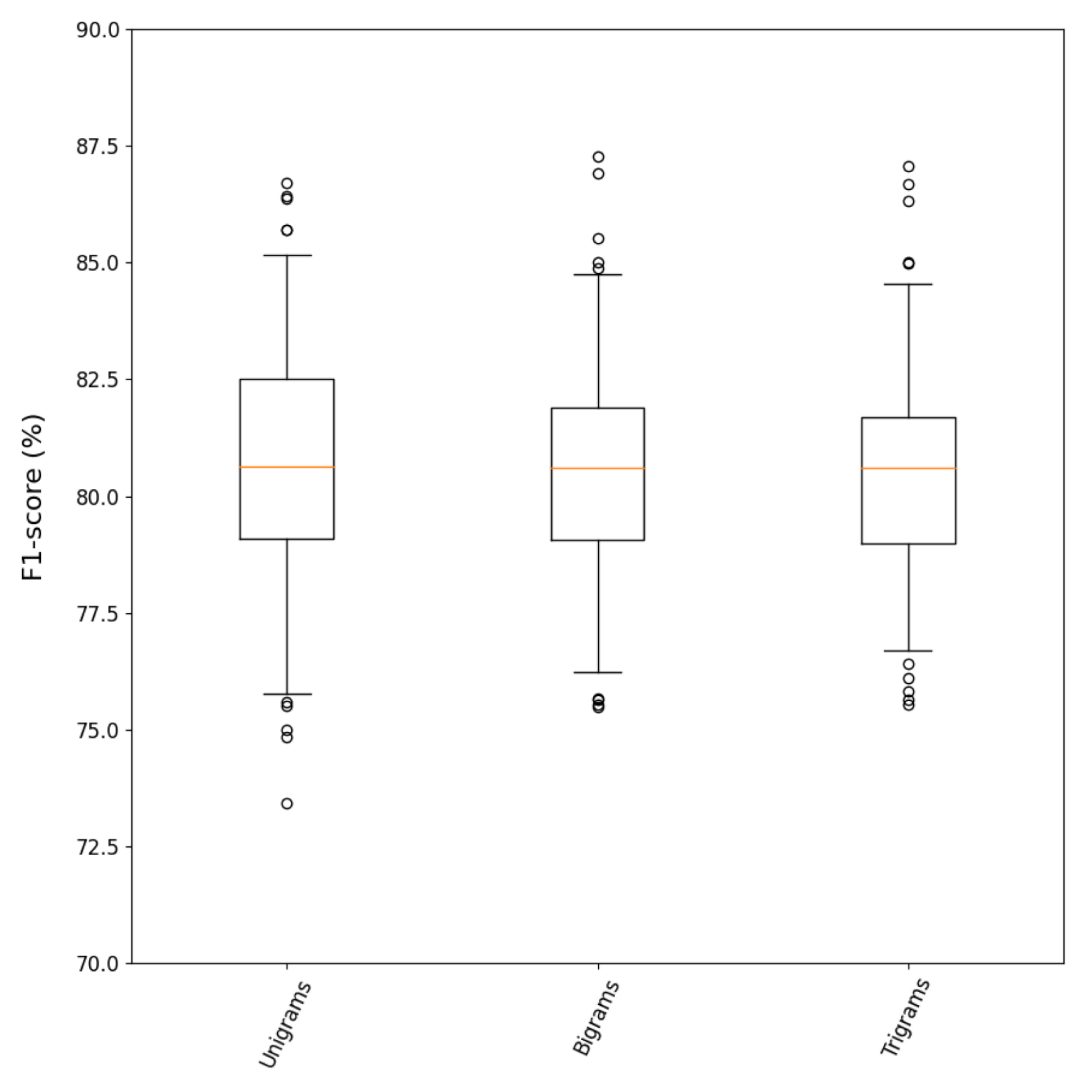
Distribution of *F*_1_ scores for the *n-grams* analysis after 200 bootstrap iterations.

The results of using bigrams and trigrams for BoW encoding are discussed in this section and displayed in Table 4 and Figure 4. The unigrams pipeline presented in Table 4 and Figure 4 is the same as the *Optimal Fields* from the previous section.

**Table 4.** Summary table with performance metrics reported as median (95% CI) and *F*_1_ interquartile variance (IQV) after 200 bootstrap iterations. The performance metrics are compared across pipelines using different n-grams from the optimal fields.

Pipeline	Precision (%)	Recall (%)	*F*_1_ (%)	*F*_1_ IQV
Unigrams	80.1 (73.9,86.0)	**82.3 (74.1,88.6)**	**80.6 (75.8,85.2)**	9.4
Bigrams	79.9 (72.2,86.9)	81.6 (74.1,88.0)	**80.6 (76.2,84.8)**	8.6
Trigrams	**80.4 (74.4,86.3)**	81.0 (73.4,88.0)	**80.6 (76.7,84.6)**	7.9

Using bigrams or trigrams did not exhibit better performance than only using unigrams. Even though multiple PK parameters are expressed with more than one term (e.g. volume of distribution, maximum concentration, area under the curve, systemic clearance), bigram and trigram features did not provide additional discriminant information. This might be caused by the high diversity on PK parameter mentions, which results in very sparse BoW representations that do not benefit from n-grams. It is noteworthy that including n-grams highly impacts the number of features in the input document representations. Therefore, it is considered that the class information that bigram and trigram features might provide does not outweigh their cost in increasing the sparsity of the occurrence matrices.

### Distributed representations

**Figure 5.**
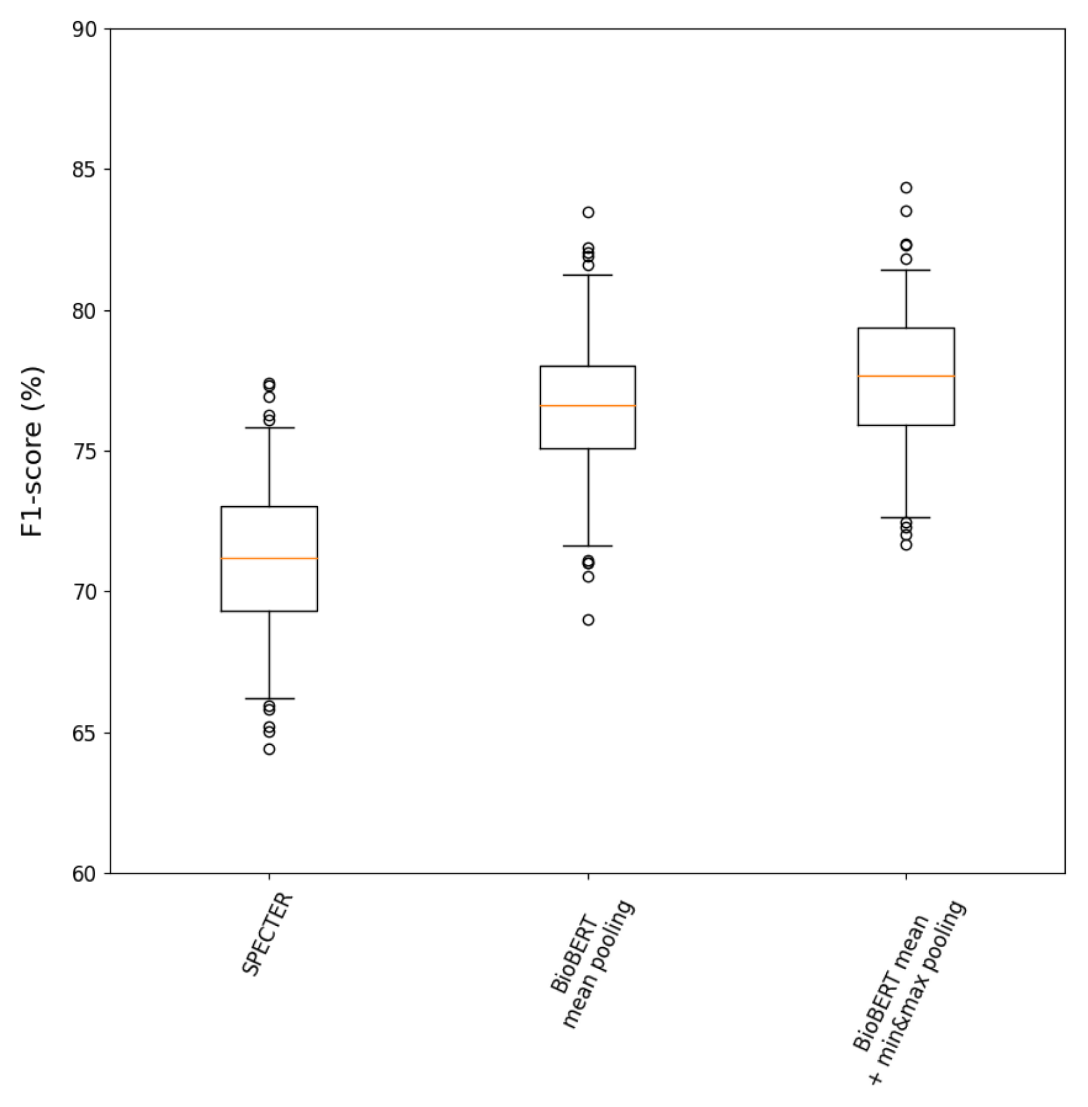
Distribution of *F*_1_ scores for the *distributed* analysis after 200 bootstrap iterations.

The effect of representing documents with word and document embeddings was studied in this section. The results are presented in Table 5 and Figure 5. Document representations obtained through SPECTER exhibited worse *F*_1_ scores than those obtained pooling BioBERT embeddings from the title and abstract tokens, with over 5% difference in the median *F*_1_ score. Although SPECTER representations have shown state-of-the-art performance for multiple document-level tasks ^39^, pooling strategies at the token-level might be better suited for this specific task since they are likely to identify whether specific terms (e.g. PK parameters) appeared in the document.

**Table 5.** Summary table with performance metrics reported as median (95% CI) and *F*_1_ interquartile variance (IQV) after 200 bootstrap iterations. The performance metrics are compared across pipelines using different distributed document representations.

Pipeline	Precision (%)	Recall (%)	*F*_1_ (%)	*F*_1_ IQV
SPECTER	74.1 (66.5,80.9)	69.0 (62.0,76.6)	71.2 (66.2,75.8)	9.6
BioBERT mean pooling	78.1 (69.0,85.4)	75.3 (68.3,82.9)	76.6 (71.6,81.3)	9.7
BioBERT mean + min&max pooling	**80.1 (71.8,86.0)**	**75.9 (69.6,82.9)**	**77.7 (72.7,81.4)**	8.7

When using BioBERT representations, it was observed that including min and max pooling resulted in better performance than only using the mean across tokens. Suppose only a small number of keywords (e.g. PK parameters) contributed to the final predictions. In that case, min&max pooling is likely to help identify the presence of those terms by extracting the most salient features from every token-embedding dimension and improve the classification performance ^42^. However, since these keywords’ appearance might also be detected with BoW approaches, in the following analyses, the effect of joining both (1) BoW + BioBERT mean pooling and (2) BoW + BioBERT mean + min&max pooling was studied.

### Final pipeline

**Figure 6.**
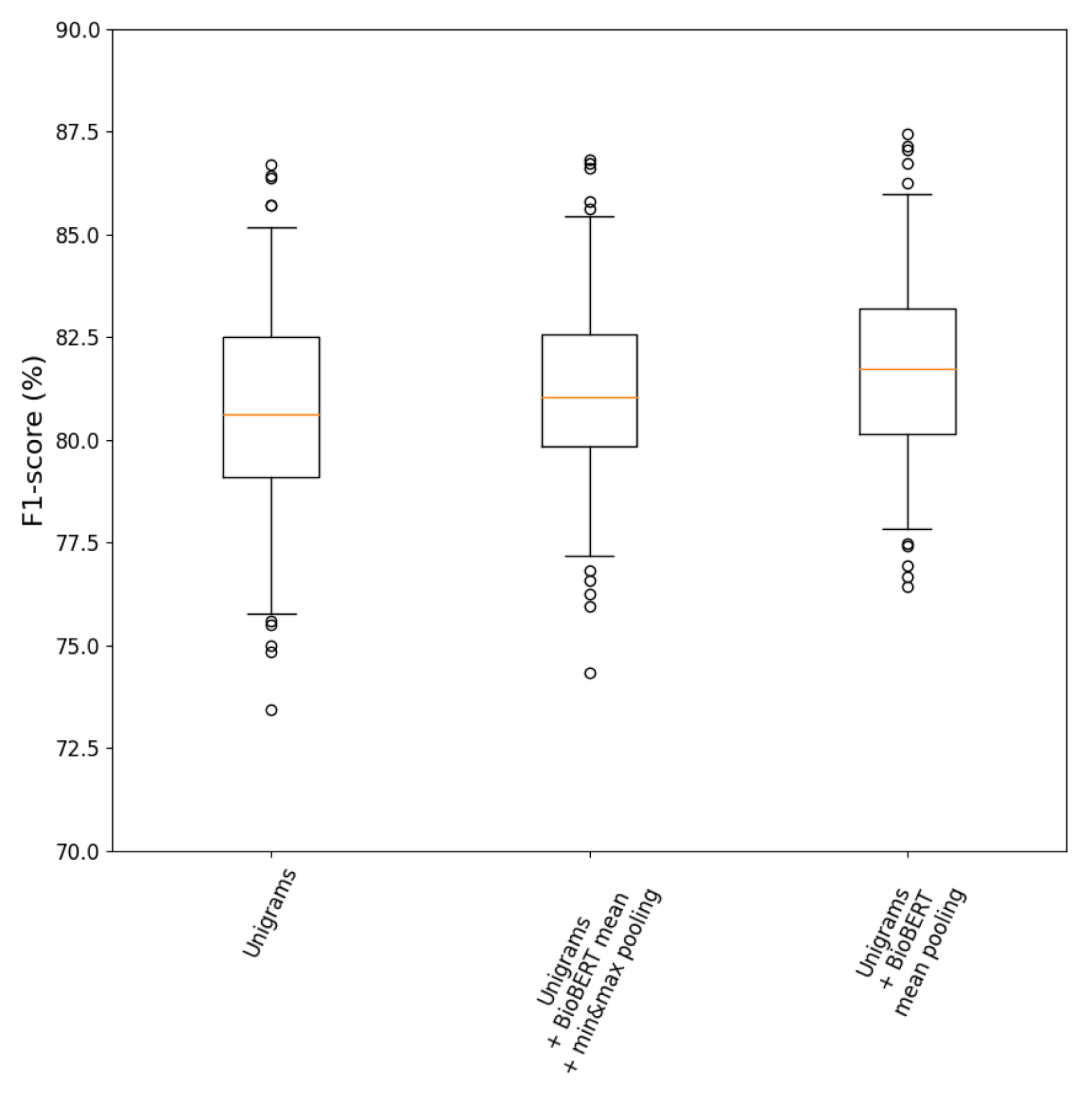
*F*_1_ score distributions for the pipelines using unigrams together with BioBERT embeddings.

The results of adding BioBERT mean pooling and BioBERT mean + min&max pooling to the unigram representations are displayed in Table 6 and Figure 6. Adding BioBERT embeddings to the unigram representations exhibited higher median *F*_1_ scores than using unigrams alone. Additionally, mean pooling across abstract and title token embeddings reported better performance than mean + min&max pooling. This suggests that the unigrams of BoW already represented the additional discriminant information provided by min&max pooling of BioBERT embeddings.

**Table 6.** Summary table with performance metrics reported as median (95% CI) and *F*_1_ interquartile variance (IQV) after 200 bootstrap iterations. The performance metrics are compared across pipelines using BoW together with distributed representations.

Pipeline	Precision (%)	Recall (%)	*F*_1_ (%)	*F*_1_ IQV
Unigrams	80.1 (73.9,86.0)	**82.3 (74.1,88.6)**	80.6 (75.8,85.2)	9.4
Unigrams + BioBERT mean pooling	83.7 (76.7,89.1)	80.4 (74.1,87.3)	**81.7 (77.8,86.0)**	8.2
Unigrams + BioBERT mean + min&max pooling	**83.8 (75.6,88.8)**	79.1 (73.4,85.4)	81.0 (77.2,85.4)	8.2

A five-fold CV approach was applied to the whole *training set* to select the pipeline hyperparameters ( Figure 2) using unigrams + BioBERT mean pooling to encode documents. The optimal hyperparameters were *min_df*=128, *max_depth*=4, *colsample_bytree*=1., which obtained a mean *F*_1_ score of 83.8% across the five folds. Finally, all the *training set* was used to fit the pipeline with the optimal hyperparameters, and it was applied to the 800 documents from the *test set*. The final metrics are reported in Table 7. The best estimates of the classifier performance on unseen data exhibited an *F*1 of 83.8% on the classification of *Relevant* publications, and an overall 93.2% accuracy across all predictions ( *Relevant* and *Not Relevant*). Wang *et al.* ^7^ developed an entity template to identify scientific publications containing PK parameters of midazolam in healthy human volunteers and achieved an *F*_1_ of 78.1%. However, the relevance criteria considered in this study was much more broad than Wang *et al.* ^7^, i.e. multiple drugs, species, conditions and study designs. Hence, the performance obtained in this study was considered to be highly effective at detecting relevant literature reporting *in vivo* PK parameters.

**Table 7.** Performance metrics of the final pipeline on the *test set*.

Precision (%)	Recall (%)	*F*_1_ (%)	Accuracy (%)
84.8%	82.8%	83.8%	93.2%

A qualitative evaluation of the classifier predictions on the *test set* was performed to detect misclassification causes. The main causes of misclassification of *Not Relevant* papers (limiting the pipeline’s precision) were: (1) papers reporting PK results of endogenous substances (e.g. insulin) considered *Relevant* and (2) physiologically-based or *in silico* PK studies reporting estimated parameter values in the abstract. We believe that the main reason for these studies’ misclassification is the highly similar frequency of critical terms to the *Relevant* papers, often reporting PK parameter values and demographic information in the abstract. Misclassification of *Relevant* papers (limiting the pipeline’s recall) was mostly observed in: (1) publications without abstract availability in PubMed, (2) PK publications with parameters mentioned in the full-text but not in the abstract and (3) animal PK studies. Cases 1 and 2 are highly difficult to identify since determining their relevance often required information from the full text. However, our observations suggest that there is still space for improvement in the detection of animal studies reporting *in vivo* PK parameters, some of which are still missed by the classifier developed in this study. It is expected that releasing this labelled corpus will encourage the testing and development of additional document classification pipelines to accelerate ADME datasets’ curation.

### Large-scale application

More than 121K publications were classified as *Relevant* when applying the pipeline to the corpus resulting from the PubMed search ’pharmacokinetics’ (n *>* 550K) in January 2021. All the *Relevant* publications were characterised by the chemicals, species and diseases mentioned in the abstract using BERN ^41^. Finally, all the papers reporting *in vivo* PK parameters were released at https://app.pkpdai.com/. The interface uses elastic search to find publications reporting *in vivo* PK parameters for specific drugs, species and conditions. Additionally, the classification pipeline was scheduled to retrieve newly published PK publications by running weekly updates. Overall, the interface provides a centralised repository of articles reporting PK parameters that researchers can use to compare and efficiently find relevant PK results.

## Conclusions

This article introduced a classification pipeline to detect scientific publications reporting *in vivo* PK parameters. By applying this pipeline to a large corpus of pharmacometric literature, we released a web resource with over 121K relevant publications to facilitate the search and comparison of PK results. Unigram features combined with mean pooling of BioBERT embeddings were found to be the optimal document representations, obtaining an *F*_1_ score of 83.8% on the test set. All the labelled data and models were released openly available to the research community on GitHub.

It is expected that this automated, open-access repository accelerates ADME dataset curation, facilitates subsequent text mining tasks and provides a centralised resource for the search of PK data, enhancing the comparison and reproduction of PK results.

## Data availability

Zenodo: PKPDAI/PKDocClassifier: PKDocClassifier. http://doi.org/10.5281/zenodo.4646953 This project contains the following underyling data:

- "training_labels.csv"
- "test_labels.csv"

Each CSV has two main columns: "pmid" and "label" containing the PubMed identifier of each publication together with the associated label (Relevant/Not Relevant), respectively.

Labels are available under the terms of the Creative Commons Zero "No rights reserved" data (CC0 1.0 Public domain dedication).

## Software availability

- Source code are available from: https://github.com/PKPDAI/PKDocClassifier
- Archived source code at time of publication: 10.5281/zenodo.4646953
- License: MIT
