# Supplementary material for: An automated approach to identify scientific publications reporting pharmacokinetic parameters
Source: Wellcome Open Res. 2021 Apr 21;6:88. [Version 1] doi: 10.12688/wellcomeopenres.16718.1 (PMC8343403; doi:10.12688/wellcomeopenres.16718.1)
Supplement: Peer review history

# Peer review: Reviewer response for version 1

**DOI:** 10.21956/wellcomeopenres.18437.r44992

**Published:** 2021-08-05

**Authors:** Dinesh Kumar Barupal

**Affiliations:** 1 Department of Environmental Medicine and Public Health, Icahn School of Medicine at Mount Sinai, New York, NY, USA

The paper describes a systematic machine learning approach to identify relevant publications which may have pharmacokinetic data. 

 I have one minor comment:

- Conventional way to find similar papers is to use the Term Frequency - Inverse Document Frequency (TF-IDF) matrix. Can the authors provide a comparison of their approach against a TF-IDF approach to find relevant papers using the training dataset (4,792 papers)?

Is the rationale for developing the new method (or application) clearly explained?

Yes

Is the description of the method technically sound?

Yes

Are the conclusions about the method and its performance adequately supported by the findings presented in the article?

Yes

If any results are presented, are all the source data underlying the results available to ensure full reproducibility?

Yes

Are sufficient details provided to allow replication of the method development and its use by others?

Yes

Reviewer Expertise:

text mining and chemoinformatics

I confirm that I have read this submission and believe that I have an appropriate level of expertise to confirm that it is of an acceptable scientific standard.

# Peer review: Reviewer response for version 1

**DOI:** 10.21956/wellcomeopenres.18437.r43730

**Published:** 2021-06-04

**Authors:** Ioannis Korkontzelos

**Affiliations:** 1 Department of Computer Science, Edge Hill University, Ormskirk, UK

This paper presents a machine learning-based approach that can automatically identify scientific publications that report in vivo pharmacokinetic parameters. The method takes as input the title and abstract of a publication and also metadata, such as author names, publication journal, keywords, MeSH terms and others.

 The paper is very well written and organised. It is easy to follow.

 The creation of the annotated dataset is described in detail, with a special focus in the inter-annotator agreement analysis.

 Experiments describe the process of evaluating the contribution of each feature/information source, to identify the best performing combination. A suggestion for improvement, or extension of the evaluation, is to consider more classification models. Currently, XGBoost is used and the number of weak estimators is a tuneable parameter.

 The conclusions are well presented by the experimental results.

 Typo:

 [In the abstract] "pipeline.In", space missing

Is the rationale for developing the new method (or application) clearly explained?

Yes

Is the description of the method technically sound?

Yes

Are the conclusions about the method and its performance adequately supported by the findings presented in the article?

Yes

If any results are presented, are all the source data underlying the results available to ensure full reproducibility?

Yes

Are sufficient details provided to allow replication of the method development and its use by others?

Yes

Reviewer Expertise:

natural language processing, text mining, machine learning

I confirm that I have read this submission and believe that I have an appropriate level of expertise to confirm that it is of an acceptable scientific standard.
